# Fabrication of Zwitterionized Nanocellulose/Polyvinyl Alcohol Composite Hydrogels Derived from Camellia Oleifera Shells for High-Performance Flexible Sensing

**DOI:** 10.3390/polym17141901

**Published:** 2025-07-09

**Authors:** Jingnan Li, Weikang Peng, Zhendong Lei, Jialin Jian, Jie Cong, Chenyang Zhao, Yuming Wu, Jiaqi Su, Shuaiyuan Han

**Affiliations:** 1 State Key Laboratory of Woody Oil Resources Utilization, Northeast Forestry University, Harbin 150040, China; lijingnan@nefu.edu.cn (J.L.); weikangp@nefu.edu.cn (W.P.); leizhendong@nefu.edu.cn (Z.L.); jjl1677292503@163.com (J.J.); congjie@nefu.edu.cn (J.C.); zcy61580909@163.com (C.Z.); wuyuming@nefu.edu.cn (Y.W.); 2 College of Material Science and Engineering, Northeast Forestry University, Harbin 150040, China; 3 Engineering Research Center of Advanced Wooden Materials, Ministry of Education, Northeast Forestry University, Harbin 150040, China; 4 Key Laboratory of Biobased Material Science and Technology, Ministry of Education, Northeast Forestry University, Harbin 150040, China

**Keywords:** cellulose-based atrp initiator, nanocellulose extraction, zwitterionized nanocellulose, ATRP, cellulose-based hydrogels, flexible sensor

## Abstract

To address the growing demand for environmentally friendly flexible sensors, here, a composite hydrogel of nanocellulose (NC) and polyvinyl alcohol (PVA) was designed and fabricated using *Camellia oleifera* shells as a sustainable alternative to petroleum-based raw materials. Firstly, NC was extracted from *Camellia oleifera* shells and modified with 2-chloropropyl chloride to obtain a nanocellulose-based initiator (Init-NC) for atomic transfer radical polymerization (ATRP). Subsequently, sulfonyl betaine methacrylate (SBMA) was polymerized by Init-NC initiating to yield zwitterion-functionalized nanocellulose (NC-PSBMA). Finally, the NC-PSBMA/PVA hydrogel was fabricated by blending NC-PSBMA with PVA. A Fourier transform infrared spectrometer (FT-IR), proton nuclear magnetic resonance spectrometer (^1^*H-*NMR), X-ray diffraction (XRD), scanning electron microscope (SEM), transmission electron microscope (TEM), universal mechanical testing machine, and digital source-meter were used to characterize the chemical structure, surface microstructure, and sensing performance. The results indicated that: (1) FT-IR and ^1^*H* NMR confirmed the successful synthesis of NC-PSBMA; (2) SEM, TEM, and alternating current (AC) impedance spectroscopy verified that the NC-PSBMA/PVA hydrogel exhibits a uniform porous structure (pore diameter was 1.1737 μm), resulting in significantly better porosity (15.75%) and ionic conductivity (2.652 S·m^−1^) compared to the pure PVA hydrogel; and (3) mechanical testing combined with source meter testing showed that the tensile strength of the composite hydrogel increased by 6.4 times compared to the pure PVA hydrogel; meanwhile, it showed a high sensitivity (GF = 1.40, strain range 0–5%; GF = 1.67, strain range 5–20%) and rapid response time (<0.05 s). This study presents a novel approach to developing bio-based, flexible sensing materials.

## 1. Introduction

With the rapid advancement of intelligent medical and wearable devices [1,2], there is an increasing demand for flexible and high-sensitivity sensing materials in biosensing applications, such as motion monitoring and cardiac detection [3,4]. Hydrogels, as soft materials with a three-dimensional network structure [5], exhibit excellent ionic conductivity [6] and tunable mechanical properties [7], making them ideal candidates for constructing flexible biosensors [8,9,10]. Among the numerous hydrogel systems, poly (vinyl alcohol) (PVA) hydrogels exhibit significant potential for biosensing applications due to their readily available raw materials, simple preparation process, and outstanding mechanical properties [11,12]. Nevertheless, PVA hydrogels possess inherent limitations: their single physical cross-linking network makes it challenging to balance mechanical strength with toughness [13,14]. Furthermore, the absence of ion-conducting groups limits their performance in high-sensitivity sensing applications [15,16].

To overcome the performance limitations of PVA hydrogels, researchers have modified them through physical blending [17,18] and chemical cross-linking [16]. Although physical blending is convenient and straightforward, the weak interactions between additives and the PVA matrix often lead to unstable material properties. Nanoparticles can be used as reinforced fillers in physical blending. For example, Tsou C. et al. [19] prepared PVA/CNT-NZnO composite hydrogels by ultrasonic solution mixing and freeze–thaw cycles. However, the use of nanoparticles often involves the phenomenon of agglomeration [20], which can cause a significant decline in the hydrogel’s conductive properties over time. While chemical cross-linking enhances the network structure of hydrogels, the introduction of cross-linking agents can compromise their biodegradability. Zhao J. et al. [21] employed Titanium bis(triethanolamine) diisopropoxide (TE) as a cross-linking agent for the preparation of PVA hydrogels [22]. The presence of chemical cross-linkers renders hydrogels cytotoxic, produces unpleasant odors, and makes PVA non-biodegradable, adversely affecting some of its properties [23]. This presents challenges for natural degradation and makes PVA a less ideal green material. And notably, atomic transfer radical polymerization (ATRP) was used in this study for the surface functionalization of nanocellulose (NC), which has advantages compared to other processes’ commercial materials [24]. Compared with conventional chemical cross-linking methods, such as the use of toxic cross-linking agents such as glutaraldehyde, the ATRP process can significantly reduce by-product generation and lower purification costs by virtue of modular reaction control and high atom economy. In terms of feedstock cost, nanocellulose extracted from oil tea husk is free of charge, which is much lower than commercial nanocellulose (5–10 USD/g) and synthetic conductive polymers (e.g., PEDOT:PSS, 20–50 USD/g). In terms of process scalability, ATRP technology is adapted to continuous flow reactors with batch yields of 50–100 g/h per unit [25], which is comparable to the industrial productivity of polyvinyl alcohol (PVA) hydrogels. In contrast, commercially available flexible sensors based on metal nanowires (e.g., silver nanowires, 300–500 USD/g) or carbon nanotubes (100–200 USD/g) are not only expensive in terms of material cost but also face agglomeration challenges in scale-up processing. Therefore, developing a green and efficient strategy for modifying PVA hydrogels with excellent overall performance remains a critical challenge.

Nanocellulose (NC), a nanomaterial derived from biomass [26,27] with high strength and a high specific surface area, is easily modified [28,29,30], exhibits excellent mechanical properties [31], and is biodegradable [32,33], making it particularly advantageous for hydrogel reinforcement. However, the absence of conducting groups on the NC surface limits its direct application in ion sensing [34]. Thanks to the robust interaction between PVA and NC, PVA hydrogels enhanced with pure NC exhibit outstanding mechanical strength. However, the absence of effective ion transport pathways limits its ionic conductivity, making it unsuitable for applications such as motion monitoring and electrocardiogram detection. Therefore, imparting the conductivity of NC and achieving synergistic enhancement with PVA has become a key focus in overcoming the performance limitations of traditional PVA hydrogels.

In this study, a green preparation strategy for utilizing biomass waste resources to produce ionized gel enhancers was proposed. Nanocellulose was first extracted from *Oleifera Shells*, and then SBMA amphiphilic groups were grafted onto the surface of nanocellulose using atom transfer radical polymerization (ATRP) to construct an ion-conducting network. Finally, the modified nanocellulose was compounded with polyvinyl alcohol (PVA) to form a hydrogel. The resulting hydrogel demonstrated a high signal-to-noise ratio (SNR) in both sports and electrocardiogram (ECG) monitoring, providing a sustainable alternative material for flexible electronic devices and promoting the high-value utilization of agricultural and forestry waste. This strategy aims to integrate the advantages of different components to endow the hydrogel with excellent performance, providing a sustainable solution for flexible electronic devices and promoting the high-value utilization of agricultural and forestry wastes.

## 2. Materials and Methods

### 2.1. Chemicals and Materials

*Camellia oleifera* shells were offered from Hunan Qiyi Biotechnology Co., Ltd. (Jiaxing, China) Anhydrous tetrahydrofuran (THF, 99.5%), anhydrous triethylamine (TEA, 99.5%), 2-chloropropionyl chloride (98%), anhydrous *N*,*N*-dimethylacetamide (DMAc, 99.8%), dichloromethane (DCM, analytical grade), polyvinyl alcohol (PVA, Mw~88,000), and *N*,*N*,*N*,*N*,*N*-pentamethyl diethylenetriamine (PMDETA, 98%) were sourced from Shanghai Titan Technology Co., Ltd. (Shanghai, China) Sulfobetaine methacrylate (SBMA, 98%) was obtained from Shanghai Macklin Biochemical Co., Ltd. (Shanghai, China) Copper(I) bromide (CuBr, 99.5%) was acquired from Shanghai Bide Pharmatech Co., Ltd. (Shanghai, China) Acetone (analytical grade) was procured from Sinopharm Chemical Reagent Co., Ltd. (Shanghai, China) Ethanol (analytical grade), methanol (analytical grade), and acetic acid (analytical grade) were purchased from Tianjin Tianli Chemical Reagent Co., Ltd. (Tianjin, China) Ammonia solution (analytical grade) was sourced from Tianjin Fuyu Fine Chemical Co., Ltd. (Tianjin, China) Sodium chlorite (analytical grade) was obtained from Tianjin Aopu Chemical Co., Ltd. (Tianjin, China) Toluene (analytical grade) was purchased from Xilong Scientific Co., Ltd. (Guangzhou, China)

### 2.2. Extraction of Nanocellulose

A total of 15.0 g of *Camellia oleifera* shell was accurately weighed and pulverized into 80-mesh powder using a reciprocating ball mill (30 Hz, MM400, Retsch, Haan, Germany). The powder was added to a Soxhlet extractor and extracted at 120 °C for 8 h using a 2:1 solution of toluene and ethanol by volume to remove the majority of the extractables, and the solid sample was collected after filtration and separation. The lignin was removed from the samples by washing with a sodium chlorite solution for 1 h at 75 °C, adjusting with glacial acetic acid to maintain a pH 3–5 environment, and repeating the washing five times to obtain hemicellulose. The removal of the remaining hemicellulose was then accomplished by washing for 2 h at 90 °C with a freshly configured 2 wt% sodium hydroxide solution. The crude cellulose was treated in the same acidic sodium chlorite system for 1 h. After separation, it was treated with a 5 wt% sodium hydroxide solution at 90 °C for 2 h to prepare alkalized cellulose, which was then made into a 2 wt% cellulose aqueous suspension. Finally, *Camellia oleifera* shell nanocellulose was produced by ultrasonic crushing for 30 min using an ultrasonic plant cell crusher (1200 W, JY98-IIIDN, Ningbo Scientz Biotechnology Co., Ltd., Ningbo, China).

### 2.3. Chlorination Modification of Nanocellulose

**Figure 1:**
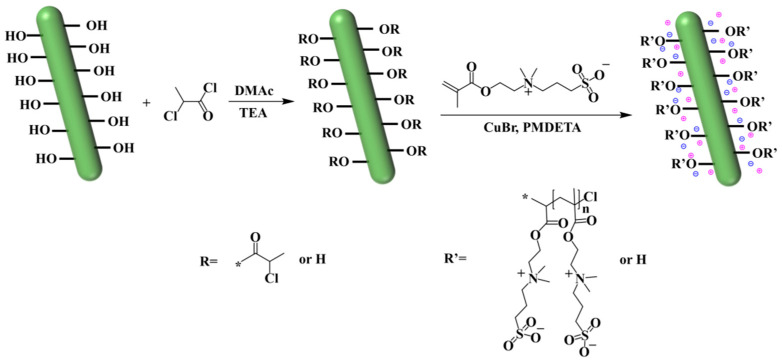
Equation for Zwitterionized Nanocellulose.

Solvent Centrifugal Replacement Method: An aqueous suspension of nanocellulose (100 g, 5.3 wt%, and 32.7 mmol) was centrifuged using acetone as the solvent at a speed of 5000 r/min. The supernatant was discarded to obtain a white transparent colloid. The centrifugation process was repeated, followed by sequential washing with tetrahydrofuran and ultra-dry tetrahydrofuran, each time centrifuging at a speed of 5000 r/min to obtain a suspension of nanocellulose in the anhydrous tetrahydrofuran system. The suspension was transferred to a reaction flask and anhydrous tetrahydrofuran was added under a nitrogen atmosphere. A total of (9.11 mL, 65.4 mmol) of anhydrous triethylamine was added while stirring, and 2-chloropropionyl chloride (8.31 mL, 65.4 mmol) was added dropwise under an ice water bath. After stirring at 40 °C for 16 h, a small amount of reaction mixture was taken and precipitated in water to obtain small brown solid particles. The precipitated liquid was centrifuged at a speed of 5000 r/min and washed sequentially with tetrahydrofuran, acetone, and distilled water to obtain a light yellow flocculent solid, which was dried as a cellulose initiator (Init-NC, 3.42 g, 15.2 mmol). The equation for Zwitterionized Nanocellulose is shown in Figure 1.

### 2.4. ATRP Polymerization

ATRP polymerization of SBMA: The Init-NC (0.377 g, 1.67 mmol) was dispersed in water to initiate the polymerization of SBMA. SBMA (4.6902 g, 21 mmol) and PMDETA (0.175 g, 1.01 mmol) were added in a reaction flask under a nitrogen atmosphere, and purified CuBr (0.24 g, 1.67 mmol) was added after 15 min, which was operated quickly, and nitrogen continued to be blown into the flask for 2–3 min to prevent the oxidation of Cu^+^. The reaction was conducted at 60 °C for 20 h 50 min to obtain a blue turbid solution. The mixture was then centrifuged at 8000 r/min, and the light blue solid precipitate was collected. This precipitate was centrifuged multiple times with distilled water, causing the blue color to gradually fade. The precipitate was taken again and washed four times using a 12.5 wt% ammonia solution as the solvent, followed by centrifugation. The precipitate turned gray-white, and vacuum filtration was performed to remove the ammonia solution, yielding the solid product. Finally, the washed solid was homogeneously dispersed in water and dialyzed using a dialysis bag with a molecular weight cut-off of 8000–14,000. The retained supernatant was also poured for dialysis. After dialysis and freeze-drying, two types of white solids were obtained, with the supernatant and precipitate labeled as PSBMA and NC-PSBMA, respectively.

### 2.5. Preparation of PVA Gels

PVA hydrogels, and all hydrogels below, were obtained by the freeze–thaw method. A total of 0.4 g of PVA was mixed with 4 mL distilled water and stirred at 95 °C for 2 h to gradually dissolve the white solid into a colorless, clear solution. The prepared aqueous PVA solution was cryogenically frozen at −25 °C and thawed at room temperature for cycling. The PVA solution was transformed into a hydrogel through physical temperature variation.

### 2.6. Preparation of PSBMA/PVA Gels

A total of 0.4 g of PVA was mixed with 7 mL distilled water and stirred at 95 °C for 2 h. As the white solid gradually dissolved, the system became colorless and transparent. Then 0.3 g of PSBMA was added to the system and stirred at 95 °C for 1 h. The prepared solution was subjected to repeated cycles of cryogenic freezing at −25 °C and thawing at room temperature. The hydrogel of PSBMA/PVA solution was formed through the physical variable of temperature.

### 2.7. Preparation of NC-PSBMA/PVA Gels

A total of 0.4 g of PVA was mixed with 7 mL of distilled water and stirred at 95 °C for 2 h. The white solid gradually dissolved and the system became colorless and transparent. Then 0.3 g of NC-PSBMA was added to the system and stirred at 95 °C for 1 h, and the solution gradually turned into a light yellow transparent state. The prepared solution was subjected to repeated cycles of cryogenic freezing at −25 °C and thawing at room temperature. The hydrogel of NC-PSBMA/PVA solution was formed through the physical variable of temperature.

### 2.8. Instrumentation and Characterization

The functional group structure of the sample was analyzed using a Fourier Transform Infrared Spectrometer (TENSOR Ⅱ, Bruker, Ettlingen, Germany) with a scanning range of 4000~500 cm^−1^, a resolution of 4 cm^−1^, and 32 scans. The sample’s structure was examined using a Nuclear Magnetic Resonance Spectrometer (AVANCE Ⅲ HD, Bruker, Zurich, Switzerland) at a frequency of 600 MHz. X-ray diffraction (40 mA, 10 kev) was used to analyze the crystalline properties of the samples using an X-ray diffractometer (XRD, ADVANCE A25, Bruker, Ettlingen, Germany). A Scanning Electron Microscope (Apreo S HiVac, Thermo Fisher, Prague, Czech Republic) was employed to observe the microscopic surface structure of the sample. Before testing, the sample was gold-sputtered, and the pores were binarized using Avizo software (Avizo2020.1) to facilitate the subsequent measurement and analysis of pore parameters. A PC-80D ECG meter (Shenzhen Carewell Electronics Co., Ltd., Shenzhen, China) was used for four-lead ECG acquisition.

An electrochemical workstation (CHI660F, Shanghai Chenhua Instrument Co., Ltd., Shanghai, China) was utilized to test the conductivity of the sample. The hydrogel sample, cut into dimensions of 10 mm × 10 mm × 1.27 mm, was clamped between two copper sheets with a thickness of 0.01 mm. The conductivity of the hydrogel sample was tested using Electrochemical Impedance Spectroscopy (EIS) in the frequency range of 10^1^~10^5^ Hz. The ionic conductivity of the hydrogel was calculated with the following Equation (1):

(1) $$\sigma =\frac{L}{R_{0}\times S}$$

where *L* was the height of the hydrogel (m); *R*_0_ was the hydrogel resistance (Ω); and *S* was the total contact area between the hydrogel and the electrode sheet (m^2^).

The stress–strain curve of the hydrogel was measured using a universal mechanical testing machine (DR-507AS, Dongguan Dongri Instrument Co., Ltd., Dongguan, China) at a speed of 50 mm/min. The sensing performance of the samples was evaluated using a digital source meter (2611B, Tektronix, Inc., Beaverton, OR, USA) with a current of 1 mA. The hydrogel sensor sensitivity was characterized by the strain sensitivity factor G_F_, which was calculated with the following Equation (2):

(2) $$GF=\frac{R-R_{0}}{R_{0}}\cdot \frac{1}{\varepsilon }$$

where *R* was the resistance of the hydrogel sensor at stretching (Ω); *R*_0_ was the initial resistance of the hydrogel sensor (Ω); and ε was the tensile strain of the hydrogel (%).

The delay time was determined by measuring the change in resistance of the compressed hydrogel at room temperature during the deformation process. The stretching distance was 2.94 mm, and the stretching speed was 100 mm/min.

## 3. Results and Discussion

### 3.1. Micro-Morphological Analysis of Camellia Oleifera Shell Nanocellulose

**Figure 2:**
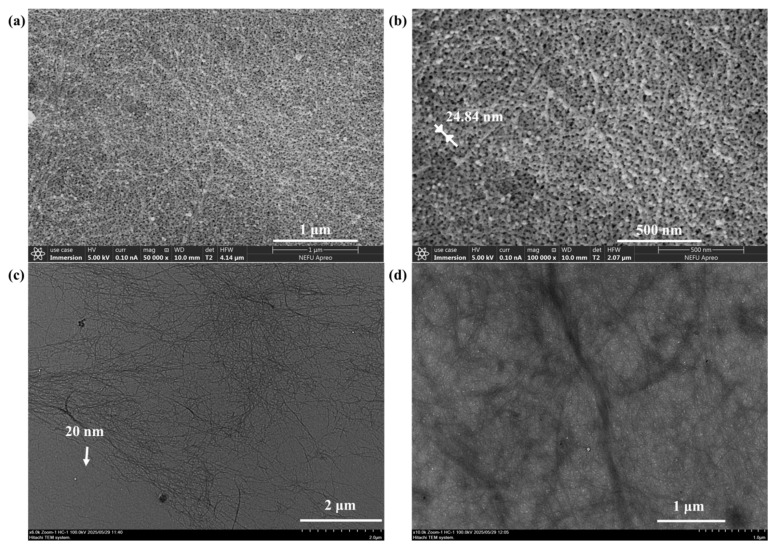
(**a**) 50,000× SEM image of NC. (**b**) 100,000× SEM image of NC. (**c**) 6000× TEM image of NC. (**d**) 10,000× TEM image of NC.

The nanocellulose extracted from the *Camellia oleifera* shells exhibited a high aspect ratio (greater than 80), with a single fiber diameter of about 24.84 nm and a length of more than 2 μm, which was in line with the size of a typical nanocellulose, which was conducive to enhancing the mechanics and network construction in hydrogels. These fibers self-assembled into cluster aggregates with widths ranging from 20 to 50 nm and lengths reaching several micrometers (Figure 2). Notably, some individual fibers protruded from the surface of the aggregate, forming a distinctive “dendritic” structure. This particular morphology offered the following advantages: on the one hand, the abundance of surface hydroxyl groups and the dendritic structure provided sufficient active sites for chemical modification, allowing functional groups to be incorporated through chlorine modification. On the other hand, the multi-level ordered structure can enhance the interfacial interaction with the PVA matrix. The improvement in the mechanical strength of the hydrogel was achieved through the physical entanglement of the fiber network and the hydrogen bonding between ionic groups and PVA hydroxyl groups.

### 3.2. Structural Characterization

**Figure 3:**
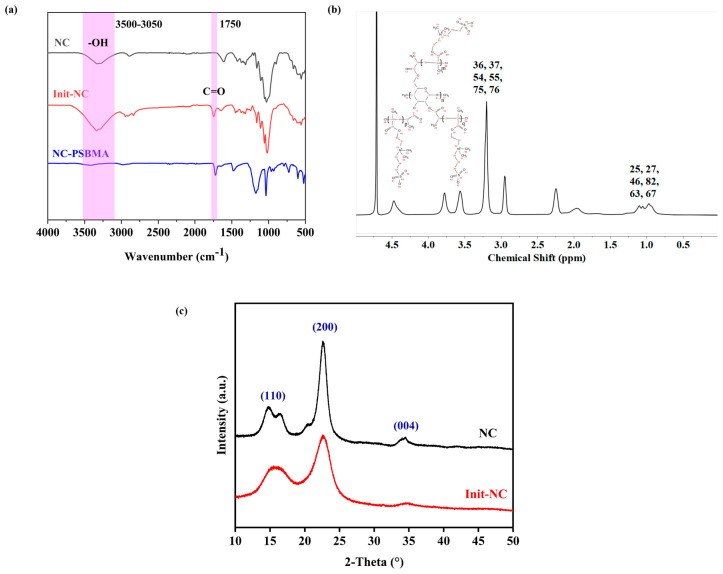
FT-IR spectrum of: (**a**) NC, Init-NC, and NC-PSBMA. (**b**) ^1^*H*-NMR spectrum of NC-PSBMA. (**c**) XRD spectrum of NC and Init-NC.

The FT-IR spectrum of freeze-dried NC and Init-NC showed (Figure 3a) a stretching vibrational peak of -CH_2_- on NC at 2890 cm^−1^. After the chlorination reaction, the Init-NC curve methylene was shifted, and the telescopic vibration peak of methyl-CH_3_ appeared in the region of 2970–2800 cm^−1^. Compared to NC, Init-NC showed a stretching vibration peak of the carbon–oxygen double bond at 1750 cm^−1^, demonstrating the successful introduction of the carbon group from 2-chloropropionyl chloride, and thereby achieving the chlorination modification of NC. Based on the FT-IR analysis above, the two-step preparation of chlorinated cellulose using the solvent centrifugal displacement method was feasible and successfully produced a chlorine-modified nanocellulose initiator.

The O-H stretching vibration peak significantly weakened in NC-PSBMA compared with NC, while the C=O stretching vibration peak at 1725 cm^−1^ was stronger (Figure 3a). This indicated that most of the hydroxyl groups on its surface were involved in forming new chemical bonds, and NC-int successfully initiated the polymerization of monomers. In the ^1^*H*-NMR spectrum of NC-PSBMA (Figure 3b), the peaks at 0.8–1.2 ppm attributed to methyl-CH_3_ (25, 27, 46, 82, 63, and 67) indicated that the number of methyl groups increased in NC-PSBMA. The peaks at 3.2–3.5 ppm attributed to the methyl group attached to N^+^ (36, 37, 54, 55, 75, and 76), and the presence of peaks of hydrogen on the cellulose carbon backbone (3.5–4.5 ppm) indicated that the NC-PSBMA was the product of the grafting polymerization that occurred. It was demonstrated that the NC-SBMA was synthesized successfully.

Both the Init-NC and the NC exhibited characteristic cellulose diffraction peaks at 2θ = 16.5°, 22.5°, and 34.5°, corresponding to the (001), (200), and (004) planes, respectively (Figure 3c). A slight decrease in the crystallinity of Init-NC compared to NC was observed, likely due to chlorination modification and swelling effects. The crystalline regions of cellulose remained largely intact, with no significant changes in crystalline morphology or adverse effects on mechanical properties.

### 3.3. Micro-Morphological Analysis of NC-PSBMA/PVA Hydrogels Surface

**Figure 4:**
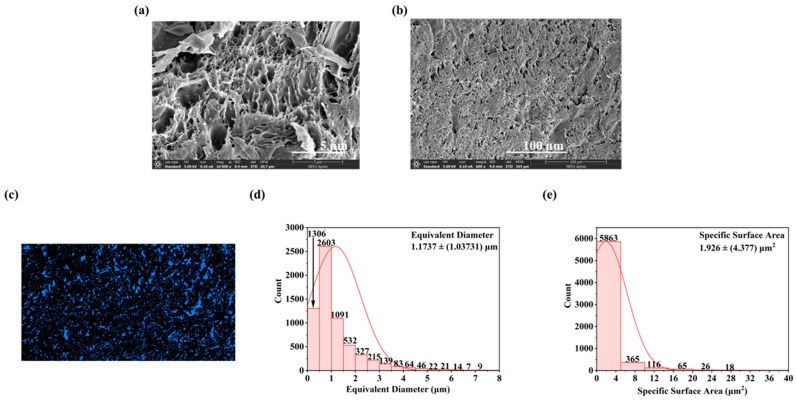
SEM images of NC-PSBMA/PVA hydrogels (**a**) 10,000× and (**b**) 600×. (**c**) Presentation effects of NC-PSBMA/PVA hydrogel pore binarization images after threshold segmentation. (**d**) Equivalent diameter distribution image of NC-PSBMA/PVA hydrogels’ pores. (**e**) Distribution image of pore-specific surface area of NC-PSBMA/PVA hydrogels.

SEM images of the NC-PSBMA/PVA hydrogel surfaces at different magnifications revealed a relatively uniformly distributed porous network at low magnification. At high magnification, the internal pore structure appeared to be non-uniformly distributed, dense, and highly interconnected (Figure 4a,b). A highly smooth hole wall structure suggested that the block fiber network of NC-PSBMA and the PVA molecular chains were fully interpenetrated through hydrogen bonding and physical entanglement. Additionally, the presence of hierarchical porous structures in certain regions further enhanced the mechanical strength of the hydrogel via the “stress dispersion effect”. This complex architecture not only promoted efficient ion transport but also simultaneously improved the tensile properties of the composite hydrogel.

Statistical analysis of the SEM images of NC-PSBMA/PVA hydrogels (Figure 4d,e) indicated an average pore size of 1.1737 ± (1.03731) μm with the size distribution from 0.253689 μm to 9.60339 μm. The specific surface area was 1.926 ± (4.377) μm^2^, and the porosity was 15.75%. This size range indicated that the pore structure was at the micrometer level, which was advantageous for the diffusion and penetration of ions or water, as well as provided sufficient spatial selectivity for ion adsorption and preventing the desorption of conductive ions.

### 3.4. Mechanical Strength Characterization of Hydrogels

**Figure 5:**
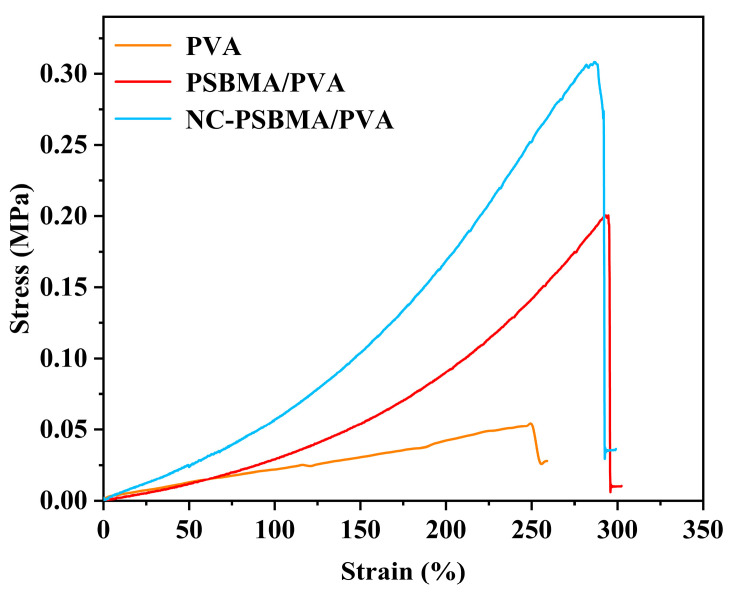
Stress–strain curve of NC-PSBMA/PVA, PSBMA/PVA, and pure PVA hydrogels.

Figure 5 presents the stress–strain curves of NC-PSBMA/PVA, PSBMA/PVA, and pure PVA hydrogels. All three exhibited similar profiles characterized by elastic deformation regions followed by strain hardening without a distinct yield point, indicating that deformation is primarily elastic throughout the stretching process. Among them, the NC-PSBMA/PVA hydrogel exhibited a narrower elastic deformation range, primarily due to the rigid, brush-like architecture of NC-PSBMA and its strong interactions with the PVA matrix, which resulted in an earlier onset of strain hardening. Nevertheless, this hydrogel achieved a remarkable elongation at a break of approximately 300%, demonstrating excellent stretchability. In comparison, pure PVA hydrogel remained in the elastic region up to failure but exhibited a lower elongation at break, with only 250% elongation, attributed to weaker intermolecular interactions. Incorporating ionic polymers (PSBMA and NC-PSBMA) significantly enhanced the mechanical strength of PVA hydrogels. The addition of PSBMA increased the tensile strength by approximately 300% to 0.2 MPa. In contrast, the inclusion of NC-PSBMA—owing to its intrinsic strength and brush-like morphology—further amplified interactions with the PVA network, boosting the tensile strength by nearly sevenfold to 0.32 MPa.

### 3.5. Characterization of Conductivity for Hydrogels

**Figure 6:**
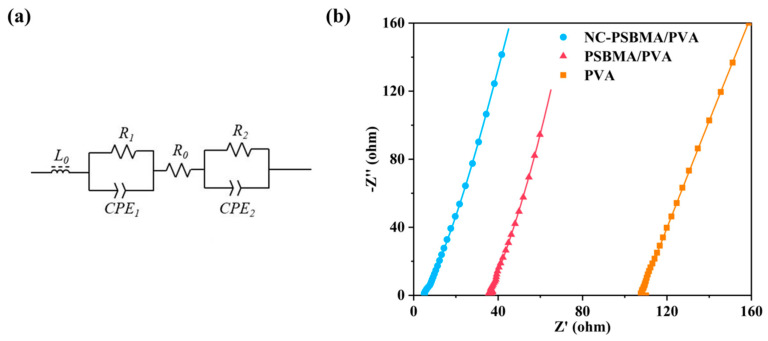
(**a**) Equivalent circuit. (**b**) Impedance spectra of NC-PSBMA/PVA, PSBMA/PVA, and pure PVA (the curves are the best fits to the equivalent circuit).

The conductivity of all samples was measured using two-electrode AC impedance spectroscopy. So, the fitted equivalent circuit model for the impedance spectra is illustrated in Figure 6a. In this model, *R*_0_ represented the intrinsic resistance of the hydrogel film. *R*_1_ and *R*_2_ corresponded to the contact resistance between the film and the two electrodes. CPE_1_ and CPE_2_ were constant phase elements, and L_0_ denoted the inductance of the overall circuit.

The impedance spectra of the NC-PSBMA/PVA, PSBMA/PVA, and pure PVA hydrogels are shown in Figure 6b. Based on the equivalent circuit fitting results and the conductivity equation in the Section 2, the conductivities of the three hydrogels were determined to be 2.652 S·m^−1^, 0.363 S·m^−1^, and 0.135 S·m^−1^, respectively. These results indicated that the incorporation of ionic polymers substantially enhanced the conductivity of pure PVA hydrogels. Notably, the conductivity of the NC-PSBMA/PVA hydrogel was 19.64 times higher than that of the pure PVA hydrogel, primarily due to the additional mobile charge carriers introduced by the ionic polymers.

Interestingly, when introduced at the same mass fraction, the conductivity of the NC-PSBMA/PVA hydrogel was approximately seven times greater than that of the PSBMA/PVA hydrogel. This enhancement cannot be explained solely by the difference in ionic group content (since PSBMA contained more ionic groups per unit mass). Instead, it was attributed to differences in molecular architecture: compared with linear PSBMA, brush-like NC-PSBMA had densely packed side chains that formed a larger steric repulsion layer within the PVA matrix, significantly improving polymer dispersibility. Moreover, its brush-like structure minimizes chain entanglement and reduces the tendency for crystallization. As a result, NC-PSBMA was more uniformly dispersed in the PVA matrix, creating more efficient ion conduction pathways and thereby dramatically enhancing the overall conductivity.

### 3.6. Sensing Performance Characterization of NC-PSBMA/PVA Hydrogel

**Figure 7:**
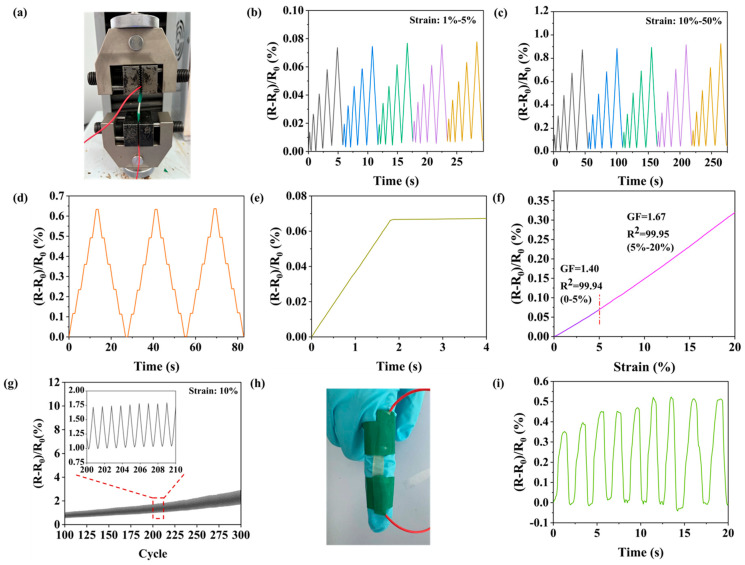
(**a**) Photograph of the testing device. Sensing resistance change curves (**b**) small strain, (**c**) large strain, and (**d**) at different strains (10%, 20%, 30%, 40%, and 50%). (**e**) Curve of resistivity with time. (**f**) Linear fitting curve of relative resistivity to strain. (**g**) Curve for 300 cycles. Sensing for finger bending, (**h**) photo of finger sensing and (**i**) curve of resistance with time.

Employing copper electrodes, the ionogel (NC-PSBMA/PVA hydrogel) was systematically evaluated as a flexible sensing detector (Figure 7a). Under continuous cyclic strain within the low-strain regime (1–5%), the material demonstrated excellent linearity, reproducibility, and high symmetry in its electrical response, as characterized by the (R − R_0_)/R_0_ curves (Figure 7b), highlighting its superior electrical sensitivity. As the strain increased to 50%, the hydrogel sensor continued to maintain a stable and reliable response curve (Figure 7c), demonstrating its effectiveness across a wide range of strains. The hydrogels maintained consistent signal outputs under fixed strains of 10%, 20%, 30%, 40%, and 50% (Figure 7d). The time-delay measurements (Figure 7e) showed that the resistance change lags behind the strain change by approximately 0.05 s, indicating a response time of 0.05 s for the hydrogel. This suggested that the ion-conducting network within the hydrogel can respond rapidly to external deformation. The gauge factors (GF) were characterized under both small-strain (≤5%) and large-strain (5–20%) conditions (Figure 7f). The GF measured 1.40 at strains below 5% and exhibited a moderate increase to 1.67 within the 5–20% strain range. This performance was comparable to that of state-of-the-art ionic conductive hydrogels, meeting the operational requirements for conventional strain sensing. During the cyclic stability experiments, the resistance of the samples showed a minor increase after 300 stretching cycles, while the resistance change sensitivity remained stable, indicating that the NC-PSBMA/PVA hydrogel sensors were suitable for repeated use and were stable for long-term use. This excellent stability may be attributed to the reversible hydrogen bonding and entangled network, which prevented structural damage during repeated deformation and ensured a consistent sensing performance. In practical applications, the hydrogel was affixed to the surface of a finger to monitor its bending motion (Figure 7h). The corresponding relative resistance change response curve (Figure 7i) showed that the peak signals exhibit distinct changes, accurately capturing the trajectory of the finger’s bending motion. These results demonstrated that the NC-PSBMA/PVA hydrogel strain sensor possessed high sensitivity, making it suitable for wearable, flexible sensors with the strong potential for real-time monitoring of human motion.

### 3.7. ECG Signal Acquisition Testing for NC-PSBMA/PVA Hydrogels

**Figure 8:**
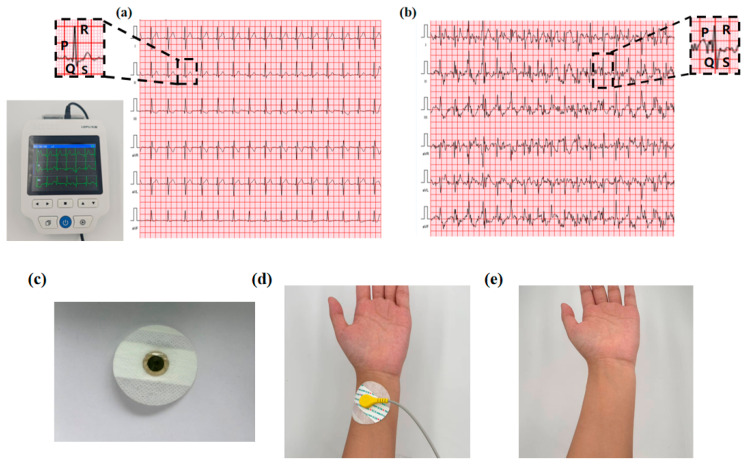
ECG acquisition signal during (**a**) rest and (**b**) motion. (**c**) NC-PSBMA/PVA hydrogel as an electrode for ECG monitoring. (**d**) Schematic diagram of the fit state of the hydrogel electrode to the skin surface. (**e**) Skin condition after 3 h of continuous wear testing.

During the ECG collection experiment, NC-PSBMA/PVA hydrogel electrodes were placed on the subject’s right arm, left arm, right leg, and left leg, and a four-lead ECG was recorded using the PC-80D ECG meter. According to the electrocardiogram, the P-wave represented atrial depolarization, while the QRS complex reflected ventricular depolarization. At rest, the waveforms exhibited regular and orderly morphology and spacing (Figure 8a), meeting the characteristics of a normal sinus rhythm, thereby confirming the signal acquisition stability of the hydrogel electrode in static conditions. During exercise, the heart rate increased, leading to a noticeable rise in the frequency of the P-wave and QRS complexes, which reflected the influence of exercise-induced stress on cardiac electrophysiological activity [35]. As shown in Figure 8b, the ECG waveform remained clearly discernible even during physical activity. This was primarily attributed to the superior skin adhesion and elasticity of the NC-PSBMA/PVA hydrogel electrode. This was attributed to the NC-PSBMA/PVA hydrogel electrodes possessing excellent skin-fitting properties. The hydrogel electrode minimized motion artifacts at the electrode–skin interface through synchronous deformation with cutaneous tissue. This conformal contact suppressed interference from relative movement, enhancing ECG signal fidelity during ambulatory monitoring. No residue adhered to the electrodes after they were peeled from the skin surface, indicating that the interfacial forces in contact with the skin were reversible, thus avoiding adhesive residue while accurately and stably monitoring the ECG. Continuous wear tests showed that the electrodes did not trigger adverse skin reactions, ensuring reliable ECG monitoring, accuracy, and safety.

## 4. Conclusions

This study successfully developed a high-performance ionic conductive hydrogel by complexing *Camellia oleifera* shell-derived and ionized nanocellulose (NC- PSBMA) with PVA. The iconic morphology of NC (diameter: ~24.84 nm, length: greater than 2 μm) enabled effective chlorination and graft polymerization to synthesize NC-PSBMA, as confirmed by FT-IR, NMR, and XRD analyses. Due to physical entanglement and hydrogen bonding, the NC-PSBMA/PVA hydrogel exhibited increased mechanical strength, with a tensile strength of 3.2 MPa (seven times higher than pure PVA) and an elongation of 300%. Optimized ion pathways enhanced ionic conductivity (2.652 S m^−1^, 19.64 times higher than pure PVA). As a flexible sensor, the hydrogel demonstrated a high sensitivity (GF: 1.4–1.67), rapid response (0.05 s), and cyclic stability (50 cycles) in strain sensing.

The excellent performance of this hydrogel all stems from the triple synergistic effect of the NC-g-SBMA and PVA composite hydrogel: (1) Amphiphilic ionic bonding enhances the cross-linking density of the polymer network through multiple hydrogen bonding and electrostatic interactions, which significantly improves the mechanical properties; (2) the formation of -SO_3_^−^/-N^+^(CH_3_)_2_ ion pairs to stable ion migration channels, which ensures the high sensitivity of the sensing performance; (3) surface charge modulation effectively reduces the contact impedance at the skin–sensor interface, which significantly improves the fit between the sensor and the skin and the signal transmission stability. As an artifact-free interference electrode, the hydrogel is capable of accurately monitoring electrocardiograms at rest and during exercise. This work not only transforms agricultural waste into advanced functional materials but also presents a sustainable design strategy for next-generation wearable bioelectronics and biomedical interfaces. In the future, we will further explore the in vivo application of hydrogels and promote the further development of hydrogels in wearable bioelectronic devices.

## Data Availability

The original contributions presented in this study are included in the article. Further inquiries can be directed to the corresponding authors.
